# Development of a Loop-Mediated Isothermal Amplification Assay Coupled With a Lateral Flow Dipstick Test for Detection of Myosin Binding Protein C3 *A31P* Mutation in Maine Coon Cats

**DOI:** 10.3389/fvets.2022.819694

**Published:** 2022-03-07

**Authors:** Pratch Sukumolanan, Kanokwan Demeekul, Soontaree Petchdee

**Affiliations:** ^1^Program of Veterinary Clinical Studies, Graduate School, Kasetsart University, Nakorn Pathom, Thailand; ^2^Department of Cardio-Thoracic Technology, Faculty of Allied Health Sciences, Naresuan University, Phitsanulok, Thailand; ^3^Department of Large Animal and Wildlife Clinical Sciences, Faculty of Veterinary Medicine, Kasetsart University, Nakorn Pathom, Thailand

**Keywords:** loop-mediated isothermal amplification, lateral flow dipstick test, myosin binding protein C3 *A31P*, mutation, Maine Coon cats

## Abstract

**Background:**

Myosin-binding protein C3 *A31P* (*MYBPC3-A31P*) missense mutation is a genetic deviation associated with the development of hypertrophic cardiomyopathy (HCM) in Maine Coon cats. The standard detection of the *MYBPC3-A31P* mutation is complicated, time-consuming, and expensive. Currently, there has been a focus on the speed and reliability of diagnostic tools. Therefore, this study aimed to develop a loop-mediated isothermal amplification assay (LAMP) coupled with a lateral flow dipstick (LFD) test to detect *MYBPC3-A31P* mutations in Maine Coon cats.

**Materials and Methods:**

Fifty-five Maine Coon cats were enrolled in this study, and blood samples were collected. *MYBPC3-A31P* was genotyped by DNA sequencing. Primers for LAMP with a LFD test were designed. The optimal conditions were determined, including temperature and time to completion for the reaction. The sensitivity of *A31P*-LAMP detection was compared between agarose gel electrophoresis (the standard method) and the LFD test. The *A31P*-LAMP-LFD test was randomly performed on seven cats (four with the *A31P* mutation and three wild-type cats).

**Results:**

The *A31P*-LAMP procedure was able to distinguish between cats with *MYBPC3-A31P* wild-type cats and *MYBPC3-A31P* mutant cats. The LAMP reactions were able to be completed in 60 min at a single temperature of 64◦C. Moreover, this study demonstrated that *A31P*-LAMP coupled with the LFD test allowed for *A31P* genotype detection at a lower DNA concentration than agarose gel electrophoresis.

**Discussions:**

This new *A31P*-LAMP with a LFD test is a successful and reliable assay with a rapid method, cost-effectiveness, and low requirements for sophisticated equipment for the detection of *MYBPC3-A31P* mutations. Thus, this assay has excellent potential and can be recognized as a novel screening test for hypertrophic cardiomyopathy associated with *MYBPC3-A31P* mutations in felines.

## Introduction

Hypertrophic cardiomyopathy (HCM) is the most common hereditary cardiovascular disease in cats (1–4). The HCM phenotype has a prevalence of approximately 15% in cats (5). Moreover, the HCM phenotype in cats is related to an increase in age. Cats with clinical HCM commonly demonstrated left-sided congestive heart failure due to an impairment of diastolic function such as respiratory distress, cardiogenic pulmonary edema, pleural effusion, syncope, hypothermia, and atrial thromboembolism (ATE) (6–9). Specific breeds are highly associated with HCM development, especially the Maine Coon (10) and Ragdoll (11).

Previous research investigated the cause of HCM in cats. Myosin binding protein C3 *A31P* (*MYBPC3-A31P*), a single nucleotide polymorphism (SNP), was possibly associated with HCM development in cats, especially Maine Coon cats (12). *MYBPC3*, the sarcomeric protein in the myocardium, plays a crucial role in heart muscle contraction. Hence, *MYBPC3-A31P* mutation disturbs normal heart function and leads to cardiac concentric hypertrophy (12, 13).

The diagnosis of the HCM phenotype routinely relies on echocardiographic measurements. While detection of the SNP missense mutation *MYBPC3-A31P* is required for genetic screening in Maine Coon cats with familial HCM. However, cats without *MYBPC3*-*A31P* mutation may develop HCM. For the detection of SNP mutations, DNA sequencing is widely recognized as the gold standard technique (14). However, due to the inconvenience of DNA sequencing, requiring labor-intensive skills, prohibitive cost, and a long time, numerous researchers have developed different methods for SNP detection, such as allele-specific loop-mediated isothermal amplification (AS-LAMP) (15), microarray genotyping (16), molecular beacon genotyping (17), allele-specific polymerase chain reaction (AS-PCR) (18), and high-resolution melting (HRM) (19). Based on advanced genetic research, LAMP is one of the accepted powerful methods for SNP detection. Applying a constant single temperature, LAMP can be used for point-of-care (POC) diagnostic tests owing to the utilization of simple instruments such as thermocyclers, heated blocks, or water baths. The advantages of LAMP are rapid detection with high accuracy because of four to six specific primers used for LAMP and isothermal amplification as a one-step process (20, 21).

In contrast, one disadvantage of LAMP is the challenge of designing a specific primer set. The detection of LAMP products is also usually performed by agarose gel electrophoresis. However, this method uses complex procedures and requires specialized equipment. The other commonly used methods for LAMP product verification are the visualization of colorimetric and lateral flow dipstick (LFD) tests, which are simple and straightforward for carrying out POC tests in a small animal hospital or small laboratory unit (22). Nonetheless, the application of LAMP combined with LFD for the detection of *MYBPC3-A31P* mutations has not yet been reported. Therefore, this study aimed to develop a specific and rapid diagnostic tool for detecting *MYBPC3-A31P* mutations in Maine Coon cats using a LAMP assay coupled with an LFD.

## Materials and Methods

### Animals and Sample Collection

Fifty-five Maine Coon cats were recruited into this study. The procedures of animal use were permitted by Kasetsart University Institutional Animal Care and Use Committee, Kasetsart University, Bangkok, Thailand (Approval number: ACKU 62-VET-059). The blood samples were collected from venous vessels for 2–3 ml per cat and stored in an ethylenediaminetetraacetic acid (EDTA) tube. The stored blood was aliquoted and kept at −20°C until DNA extraction. As described previously, the process of DNA extraction was conducted via Blood Genomic DNA Extraction Mini Kit (Favorgen, Taiwan) regarding manufacturer's recommendations (23).

### Genotyping of *MYBPC3-A31P* Mutation

The protocol for genotyping of *MYBPC3-A31P* mutation was obtained from a previous study by Godiksen et al. (24). The details of forwarding and reverse primers were represented in Table 1. In brief, the steps of PCR included heat activation at 95°C for 15 min, 35 cycles of 3 steps of (i) denaturation at 95°C for 30 s, (ii) annealing at 58°C for 30 s, and (iii) extension at 72°C for 1 min and final extension at 72°C for 10 min. The size of the PCR product was 242 bp. PCR purification was then conducted following manufacturer's protocol (Favorgen, Taiwan). The Sanger sequencing was applied to determine the nucleotide of the *MYBPC3* gene. The mutation of *MYBPC-A31P* was evaluated by using the Bioedit program. After *MYBPC3-A31P* genotyping, the enrolled cats were divided into two groups: wild-type genotype and mutant genotype. Three cats from the wild-type group and four cats from the mutant group were randomly selected, owing to the concentration and purity of DNA extraction for the detection of the *A31P*-LAMP method coupled with LFD test.

**Table 1:** Primer sets for *MYBPC3-A31P* genotyping.

**Primer**	**Sequence (5'-3')**	**Length (bp)**	**Tm (**°**C)**	**GC (%)**
*A31P* forward	AGCCTTCAGCAAGAAGCCA	19	51.1	53
*A31P* reverse	CAAACTTGACCTTGGAGGAGC	21	54.4	52
F3-*A31P*	CCATTGGCCCATCTCAGTC	19	53.2	58
B3-*A31P*	TGCGTAGGGTCCCTGGTC	18	54.9	67
FIP-*A31P*	CCTCGAACACAGCAGAGC-TCAGCCTTCAGCAAGAAGCC	38		
BIP-*A31P*	Biotin-GGCAGTGACATCAGCGCCA-CTGTCAGAGTGTGCCTCGTG	39		
*A31P*-probe	6-FAM-AGAGCGGTCAGGAGTAAAGG	20	53.8	55

*The underline represents the MYBPC3-A31P SNP area on the A31P-LAMP primer*.

### *A31P*-LAMP Primer Design

**Figure 1:**
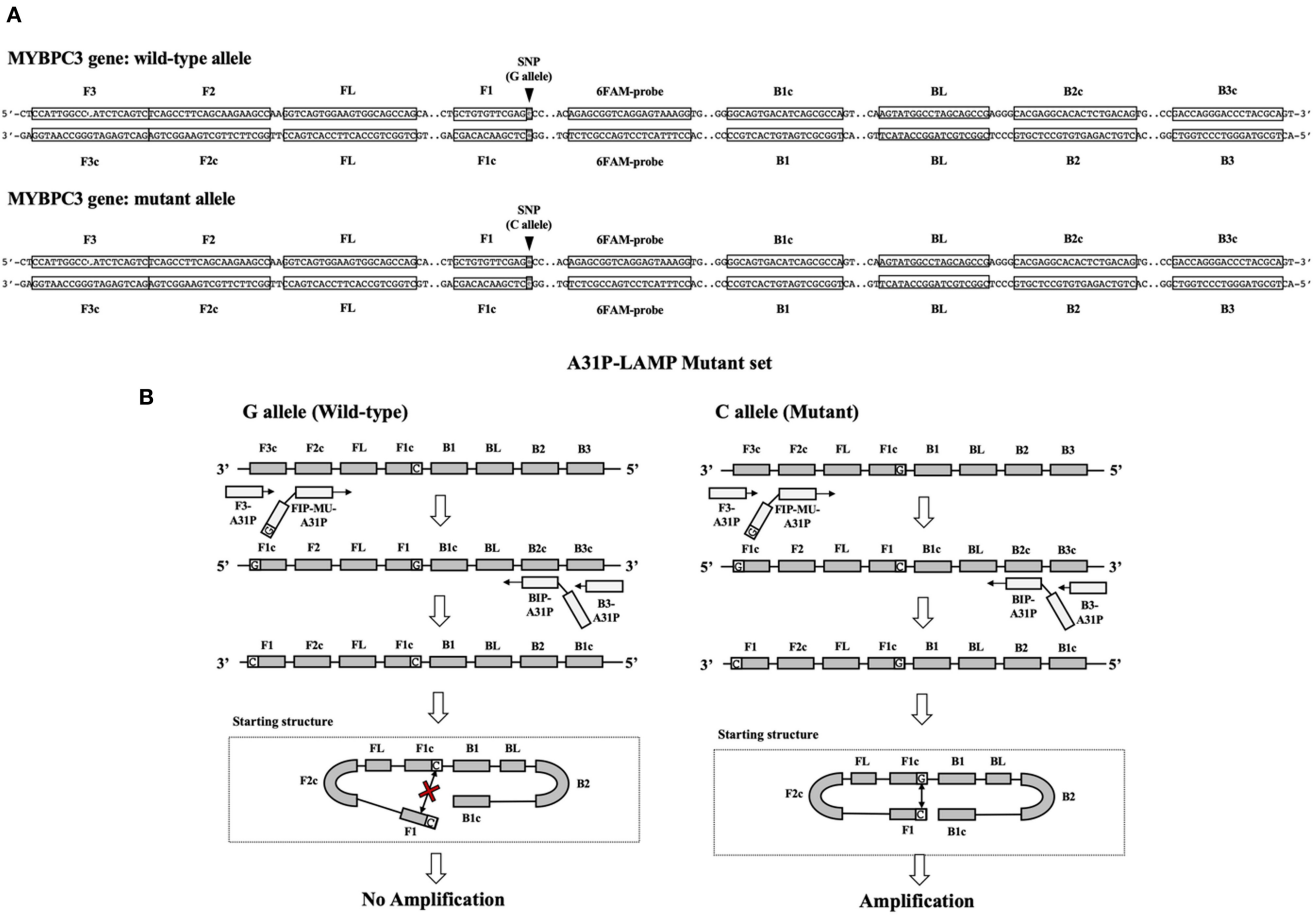
The schematic of target sequence *A31P*-LAMP for detecting the *MYBPC3-A31P* and *A31P* LAMP process. **(A)** The homozygous wild-type and homozygous mutation of the *MYBPC3-A31P* were represented. The target of the *A31P*-LAMP primer regions including outer primer (F3 and B3 primer) and inner primer (FIP and BIP) was demonstrated. The dark triangle symbols pointed to *MYBPC3-A31P* SNP. **(B)** The schematic displayed the *A31P*-LAMP process. The guanine (G) allele from the homozygous wild type was not amplified while the cytosine (C) allele from homozygous mutation was amplified.

The *A31P*-LAMP primer set was designed by using Primer Explorer version 5 software (http://primerexplorer.jp/lampv5e/index.html). Based on our experience, the high guanine and cytosine (G/C) nucleotide in the sequence of the *MYBPC3* required a manual design of the primer set. The DNA sequence of *MYBPC3* protein was obtained from the National Center for Biotechnology Information (NCBI) database. In this research study, we designed the specific primer set based on the efficiency of recognition of SNP at position *A31P* for specifically selective amplification. The four designed primers can be used to detect mutant allele, cytosine (C), at the *A31P* missense mutation point from the *MYBPC3*. Four specific primers of *A31P*-LAMP, including forwarding inner primer (*A31P*-FIP), backward inner primer (*A31P*-BIP), *A31P*-F3 primer, and *A31P*-B3 primer were designed under the consideration of correlative comparable in the number of base pairs, percentage of G-C components, and melting temperatures. The position of *MYBPC3-A31P* was located at the 5' end of the designed forward inner primer (*A31P*-FIP). The designed primers set in this study were shown in Table 1. The illustration of the *MYBPC3*, location of *A31P*-LAMP primer, and steps of the *A31P*-LAMP process are represented in Figure 1.

### *A31P*-LAMP Condition

*A31P*-LAMP solution was performed in a total volume of 25 μl per reaction. The mixture of reaction consisted of the following contents: 1X isothermal amplification buffer, containing 20 mM Tris-HCl, 10 mM (NH_4_)_2_SO_4_, 50 mM of KCl, 2 mM MgSO_4_, 0.1% Tween (New England Biolabs, United States), 6 mM of MgSO_4_ (8 mM in final concentration), 1.4 mM of dNTPs (Thermo Scientific, United States), 0.8 M of betaine (Sigma-Aldrich, United States), 7.5% of DMSO, 0.2 μM of *A31P*-F3 primer, 0.2 μM of *A31P*-B3 primer, 1.6 μM of *A31P*-FIP primer, 1.6 μM of *A31P*-BIP primer, 8 unit of *Bst* 2.0 DNA polymerase (New England Biolabs, United States), 9.4 μl of sterile water, and 2 μl of DNA template. RNase-free water was used as a negative control in each experiment. To optimize the reaction time of *A31P*-LAMP, the initial condition was modified from the manufacturer (New England Biolabs, United States). The optimal temperature of the *A31P*-LAMP reaction was evaluated at the gradient temperature, ranging from 60.8 to 68.0°C. Moreover, the mixtures of LAMP reaction were experimented with to incubate with different time points for 30, 45, 60, and 75 min.

### Lateral Flow Dipstick Test

**Figure 2:**
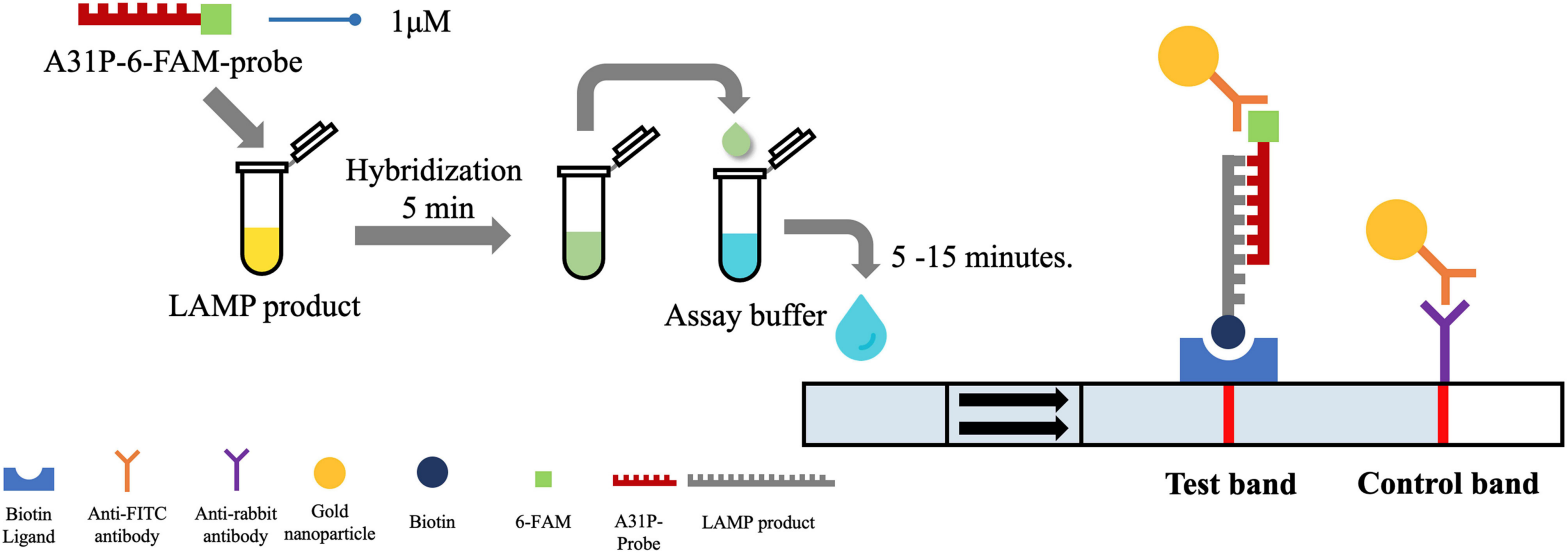
The illustration of the principle of LFD test. The *A31P*-LAMP assay coupled with the LFD test showed the positive purple-red color band in the control line. In homozygous wild-type cats, the band was not represented in the test band while the positive test band appeared in cats with the *MYBPC3-A31P* mutation.

After the *A31P*-LAMP protocol was optimized, an LFD test was conducted for validating the test efficacy. Regarding this study, the designed *A31P*-LAMP products were labeled with biotin-tagged BIP. In our experiment, an LFD test was performed according to commercial manufacture protocol (Melinia Genline HybriDetect, GieBen, Germany). In short, the amplificated *A31P*-LAMP product was added with 1 μM of *A31P*-probe which was labeled with a 6-carboxyfluorescein (6-FAM) probe and allowed to hybridize in the same temperature of *A31P*-LAMP amplification for 5 min. The sequence of designed *A31P*-probe is represented in Table 1. Then, the 10 μl of hybridized LAMP products were directly dropped on lateral flow test strips. After that, lateral flow test strips were dipped into 80 μl of assay buffer that has been rewarmed at room temperature. Subsequently, the assay solution was emigrated by chromatography method along the test strips. The results of the LFD were observed by the naked eye within 5–15 min (Figure 2). A positive result for the MYBPC3-*A31P* mutation was represented by two bands, the control line and the test line, appearing on the strip of the LFD test in a purple-red color. One band, the control line, appeared for homozygous wild-type cats. If no band appeared for the control line, the results of the LFD test were considered invalid.

### Verity of the Diagnostic Test for the *A31P*-LAMP Test

The analytical sensitivity of the *A31P*-LAMP test was determined in this study. The DNA of enrolled cats with *MYBPC3-A31P* mutation was diluted in various concentrations, including 10, 1, 0.1, 0.01, 0.001, 0.0001, and 0.00001 ng/μl. *A31P*-LAMP reaction was performed according to the protocol as described above. Then, the *A31P*-LAMP product was detected via (LFD test in comparison with a standard 1.5% agarose gel electrophoresis technique (25).

## Results

### Descriptive Demographics of the Animals

**Figure 3:**
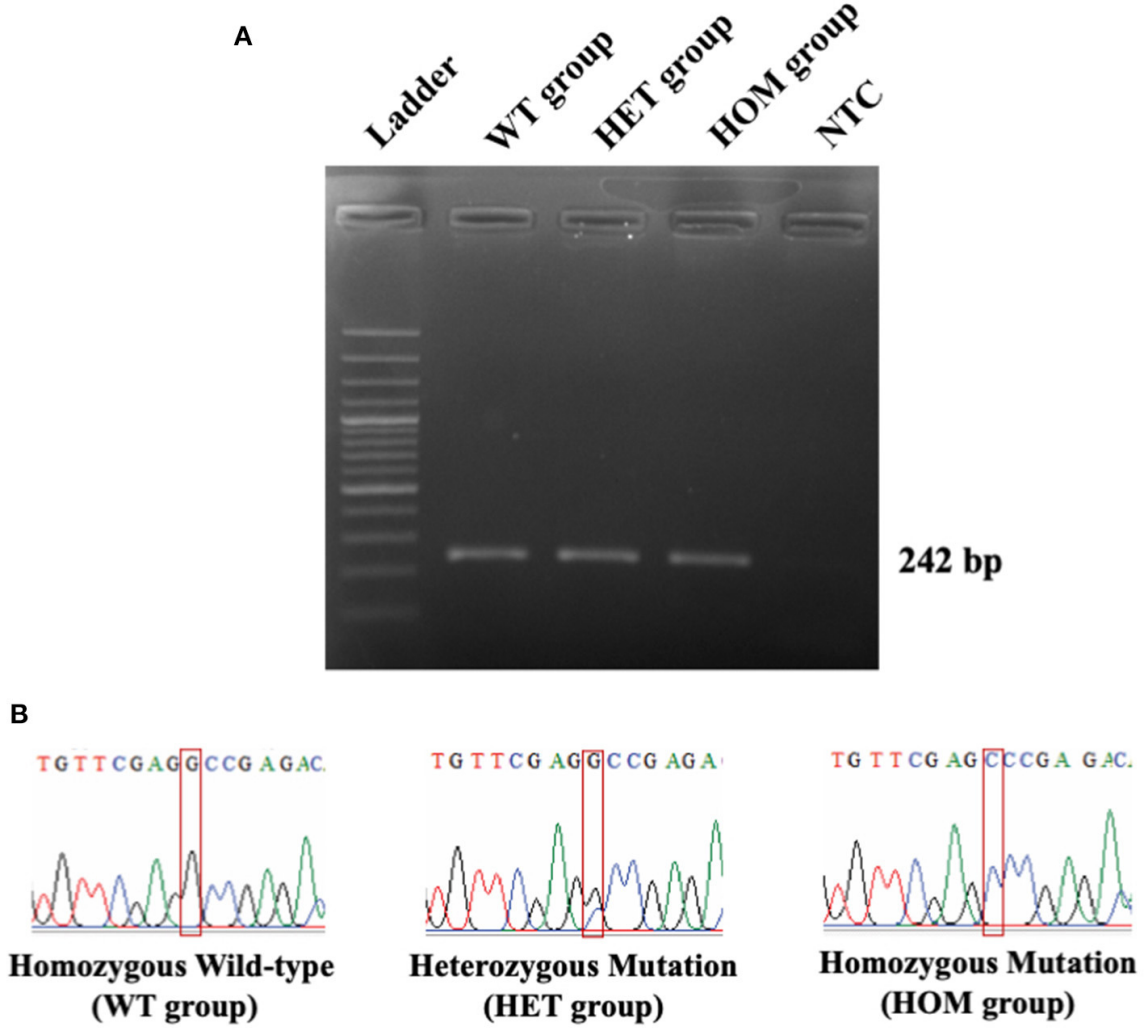
The DNA sequencing results of *MYBPC3-A31P* mutation. **(A)** The PCR result from *MYBPC3* amplification. Lane 1: DNA ladder; Lane 2: homozygous wild-type group; Lane 3: heterozygous mutation group; Lane 4: homozygous mutation group; Lane 5: negative control (NTC). The product size relied on 242 bp. **(B)** The chromatograms from the Sanger sequencing method were demonstrated in three different types of *MYBPC3-A31P* mutation. The red box represents the location of the *MYBPC3-A31P* region.

In this study, 55 Maine Coon cats were recruited to the experiment. The average age of the enrolled cats was 22.54 ± 3.22 months (mean ± SEM). Their average body weight was 5.73 ± 0.35 kg (mean ± SEM). There were 30/55 (54.55%) male and 25/55 (45.45%) female Maine Coon cats. The DNA sequencing results revealed that 8/55 cats (14.55%) had the *MYBPC3-A31P* mutation. Among these, 1/55 (1.82%) was a homozygous mutation and 7/55 (12.73%) were heterozygous mutations. The demographics of the seven randomly selected Maine Coon cats are presented in Table 2. Figure 3 represents the DNA sequencing examination of the *MYBPC3-A31P* mutation.

**Table 2:** Demographic information of animals in this study.

**Parameters**	**Overall cats** **(*n* = 55)**	***A31P*-LAMP-LFD** **(*n* = 7)**
Age (months)	22.54 ± 3.22	24.57 ± 5.94
Weight (kg)	5.73 ± 0.35	5.56 ± 0.48
Male [number (%)]	30/55 (54.55%)	4/7 (57.14%)
*MYBPC3-A31P* mutation [number (%)]	8/55 (14.55%)	4/7 (57.14%)

### Design of the *A31P*-LAMP Primer for Detecting the *MYBPC3-A31P* Mutation

**Figure 4:**
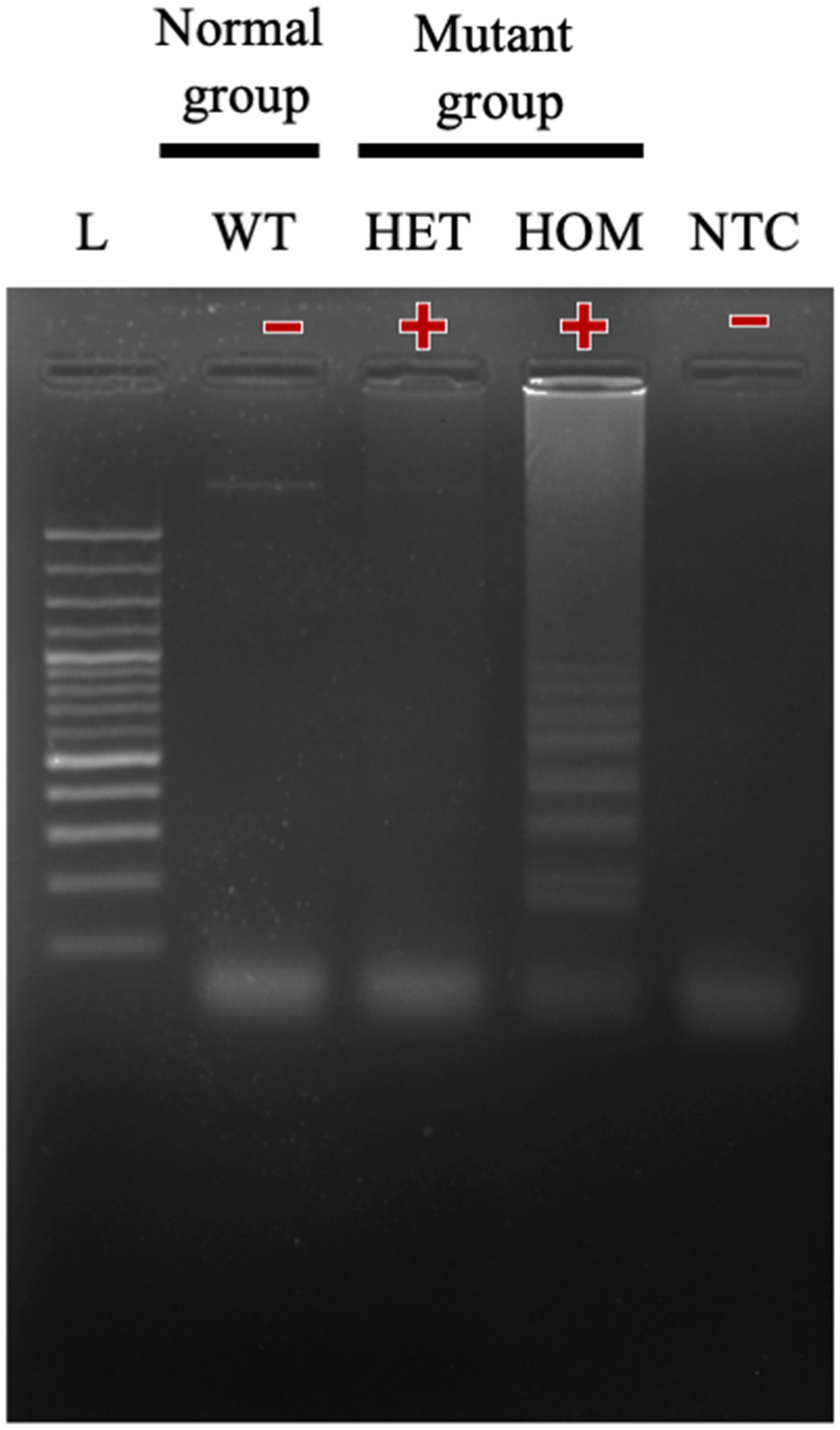
The results of the *A31P*-LAMP reaction for identifying *MYBPC3-A31P* mutation in Maine Coon cats by agarose gel electrophoresis. The results showed a positive amplified band in Lane 3 and Lane 4 from both heterozygous mutation and homozygous mutation. In Lane 1, the cats from the homozygous wild type did not amplify the *A31P*-LAMP.

In this study, we successfully designed a specific *A31P*-LAMP primer for detecting the mutant allele cytosine (C) of the *MYBPC3-A31P* mutation. *MYBPC3-A31P* is located at the 5' end of the forward inner primer (FIP) to distinguish between the wild-type genotype and mutant genotype in Maine Coon cats. The *A31P*-LAMP reaction on the *MYBPC3-A31P* mutation in cats is demonstrated in Figure 4. The mutated allele (C), including the heterozygous mutation (G/C) (Lane 3) and the homozygous mutation (C/C) (Lane 4), was amplified and evaluated via agarose gel electrophoresis as the standard method. However, homozygous wild-type (G/G) was not able to be observed by *A31P*-LAMP detection. Therefore, the designed *A31P*-LAMP primer set is an excellent LAMP primer set for identifying the *MYBPC3-A31P* mutation.

### Optimizations of the Designed *A31P*-LAMP Amplification

**Figure 5:**
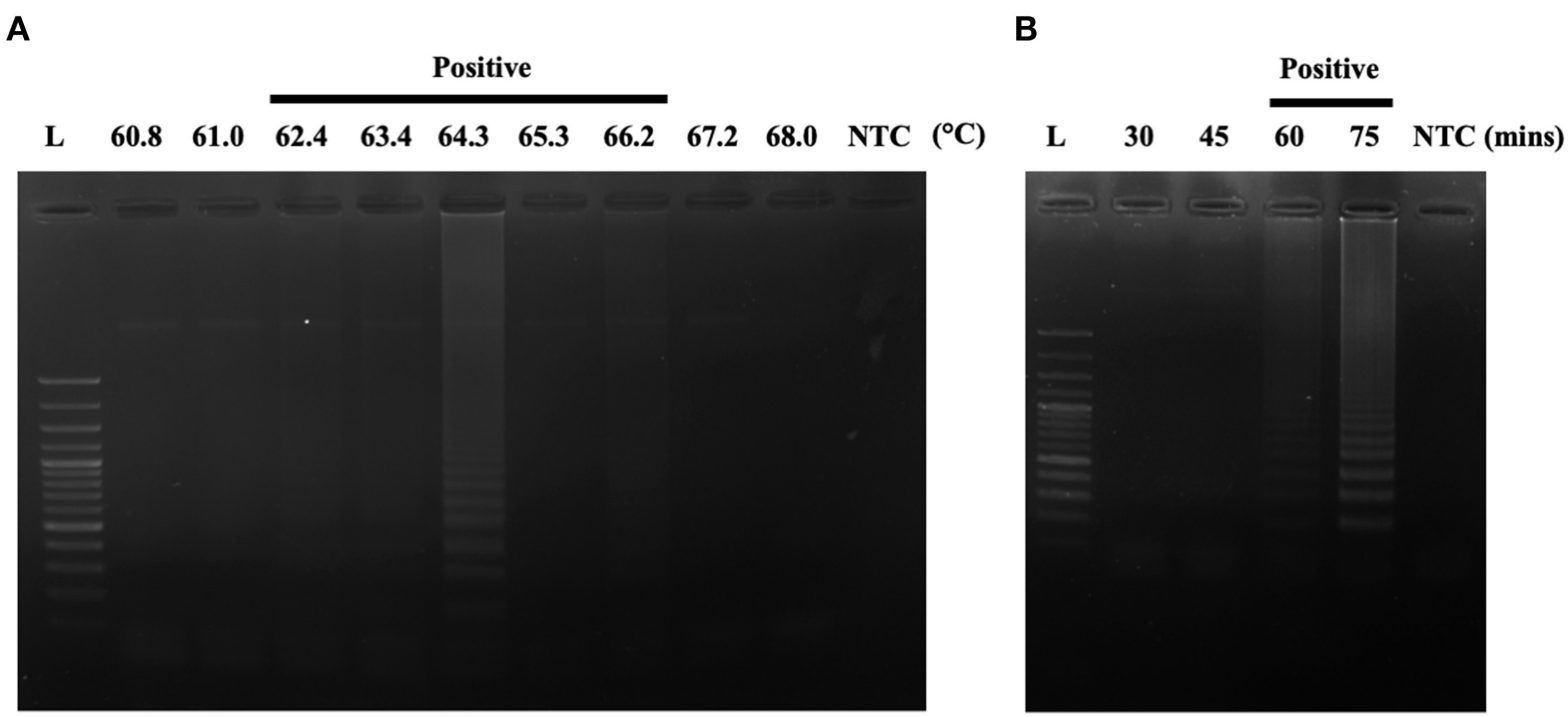
The optimization of the *A31P*-LAMP protocol. **(A)** The optimal temperature was examined from 60.8 to 68°C. **(B)** The optimal temperature was evaluated from 30, 45, 60, to 75 min. The A31P-LAMP product was demonstrated within 60 min.

After designing an effective *A31P*-LAMP primer set, we sequentially optimized the conditions of the temperature and time to perform the *A31P*-LAMP reaction. The optimal temperatures were tested from 60.8 to 68.0°C. We observed that *A31P*-LAMP was successful at 62.4 to 66.2°C (Figure 5A). Thus, we selected the temperature of 64°C as optimal for the *A31P*-LAMP reaction. Moreover, the time required for the *A31P*-LAMP reaction was optimized. We discovered that *A31P*-LAMP had positive results from 60 to 75 min (Figure 5B). Therefore, the *A31P*-LAMP reaction at 60 min provided a better experimental outcome based on the higher intensity signal on gel electrophoresis.

### Evaluation of *A31P*-LAMP Coupled With Lateral Flow Dipstick Test

**Figure 6:**
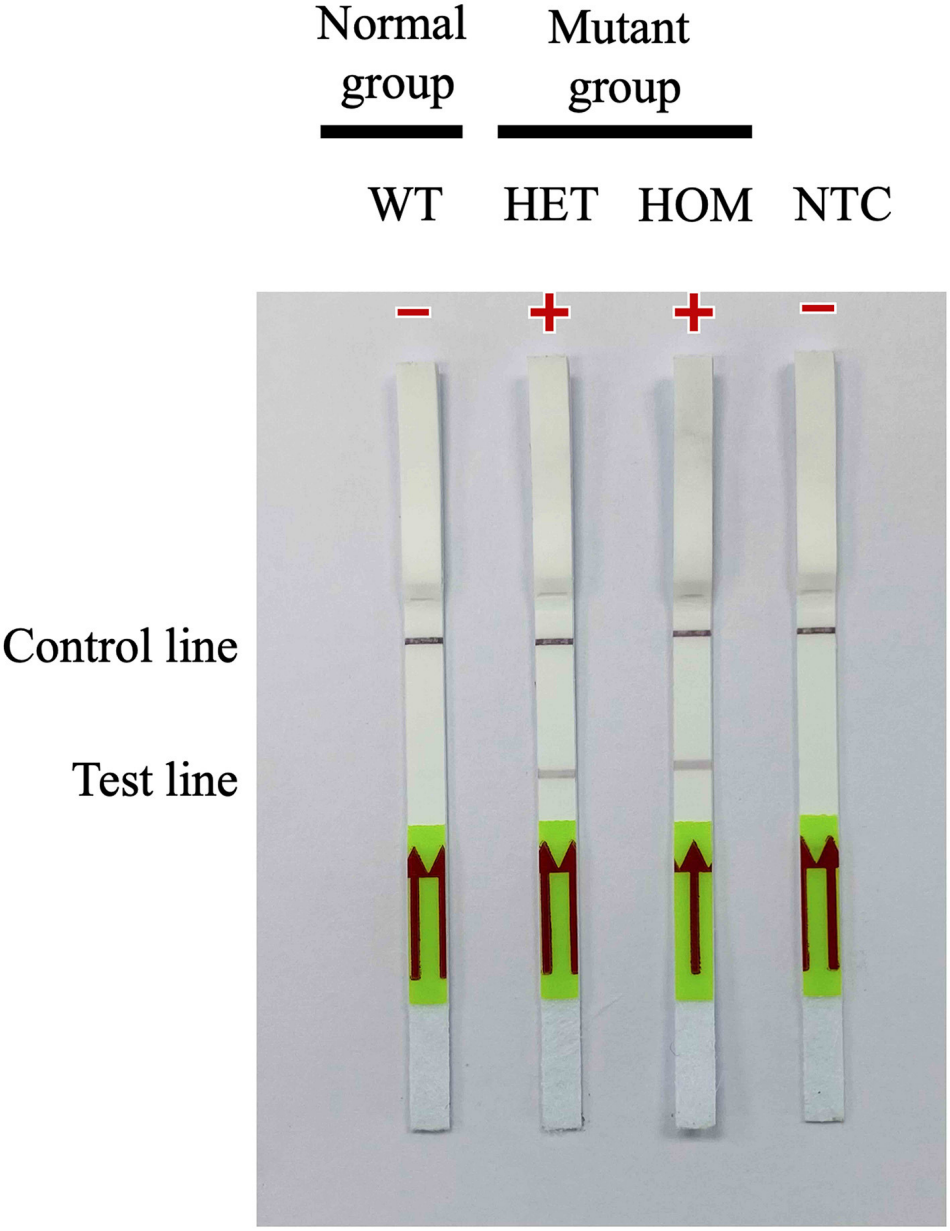
Detection of the DNA (10 ng per μL) of *MYBPC3-A31P* mutation using the recombinase polymerase amplification-LFD test. WT, wild-type cats; HET, heterozygous mutation; HOM, homozygous mutation; NTC, negative control.

*A31P*-LAMP coupled with the LFD test was operated to detect the designed LAMP products. Biotin was tagged at the backward inner primer (*A31P*-BIP) and 6-carboxyfluorescein (6-FAM) was labeled in the *A31P*-probe. In this experiment, two purple-red color bands for the test line and control line appeared for mutant cats with either a heterozygous or homozygous mutation. However, only one band appeared at the control line in homozygous wild-type cats (Figure 6). Overall observations suggested that the *A31P*-LAMP coupled with the LFD test was potentially suitable for detecting *MYBPC3-A31P*.

### Verity of a Diagnostic Test for *A31P*-LAMP Test

The sensitivity test of the designed *A31P*-LAMP reaction for detecting *A31P*-LAMP product was investigated using 10-fold serial dilutions of DNA concentration in comparison between agarose gel electrophoresis (standard method) and LFD test. In agarose gel electrophoresis, we found that the lowest DNA concentration to detect LAMP-*A31P* was 0.0001 ng/μl.

**Figure 7:**
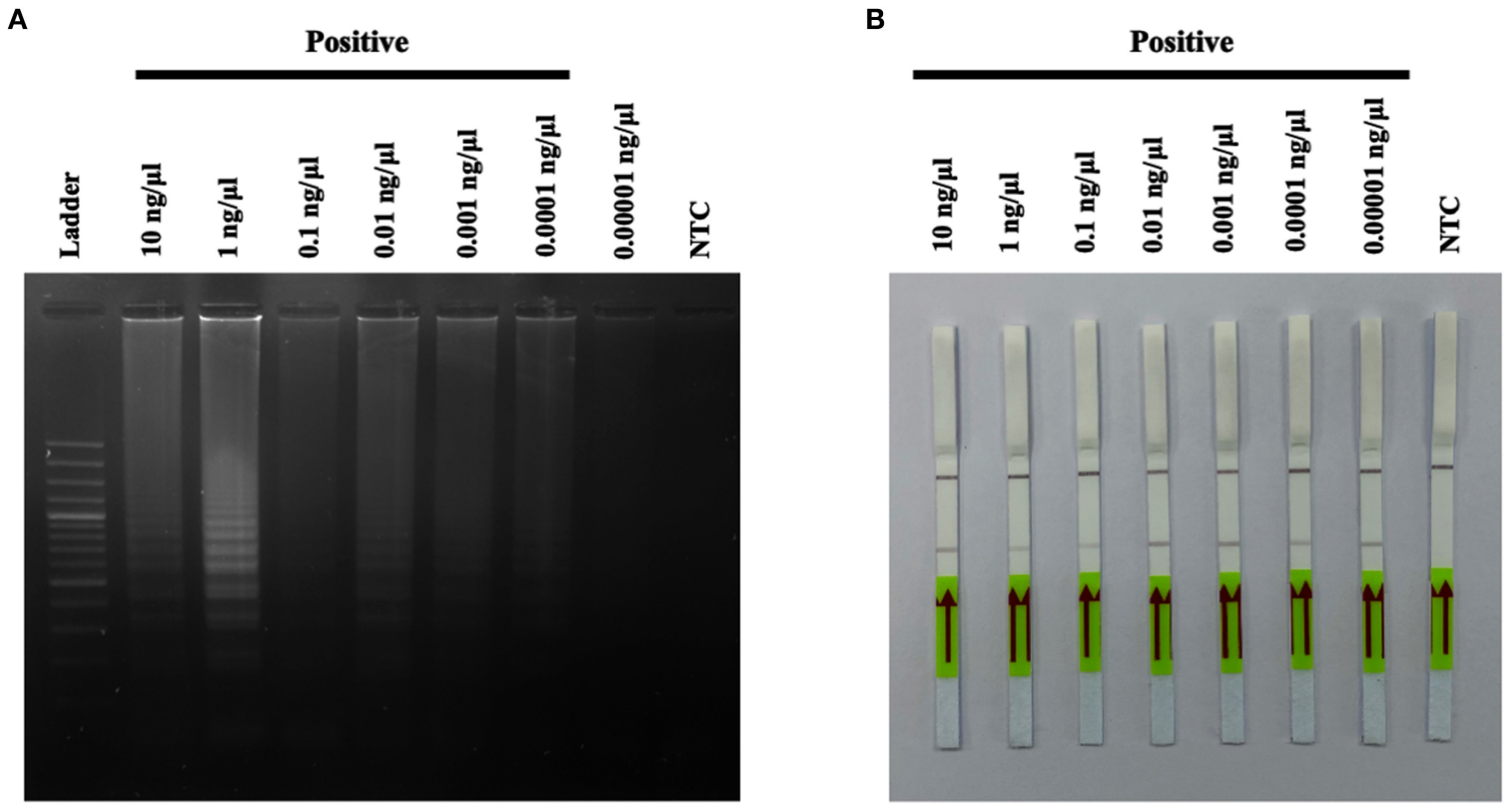
The results of *A31P*-LAMP reaction from various DNA concentrations (10–0.00001 ng per μL) for identifying *MYBPC3-A31P* mutation in Maine Coon cats by agarose gel electrophoresis **(A)**. Detection of the DNA (10–0.00001 ng per μL) of *MYBPC3-A31P* mutation using the recombinase polymerase amplification-LFD test **(B)**. NTC, negative control.

In this study, seven out of 55 cats were diagnosed using the *A31P*-LAMP assay coupled with the LFD test, all seven cats were used in various DNA concentrations (from 0.0001 to 0.00001 ng/μl) to detect *A31P* mutation. The results showed that 0.00001 ng/μl of DNA concentration is the minimal amount that was able to detect *A31P* mutation with the LFD test in all seven cats (Figure 7). The accumulated finding indicated that the LFD test provided more sensitivity to identify *A31P*-LAMP product than agarose gel electrophoresis.

## Discussion

The *MYBPC3-A31P* mutation is of great concern in Maine Coon cats due to the severity of the pathogenesis and the HCM disease progression (26). According to our findings in the recruited study animals, the *MYBPC3-A31P* mutation rate was 14.55%, which is low. In concordance with our recent prior publication, the mutation rates of *MYBPC3-A31P* and *A74T* in Maine Coon cats accounted for 21.43% in Thailand (23). The prevalence of *MYBPC3-A31P* mutations worldwide (not included in Thailand) is around 34% (27). Moreover, a study in a large European region showed that the prevalence of Maine Coon cats with the *MYBPC3-A31P* mutation was 41.5% (28).

To date, the gold standard for the detection of *MYBPC3-A31P* mutations is DNA sequencing. However, this method has various limitations, such as complicated processes, time-consuming, a prohibitive cost, and requiring special equipment. Over the past few decades, many researchers have attempted to develop better techniques for detecting SNP gene mutations. There is currently no perfect screening test that can be applied as a simple diagnostic tool for SNP gene mutations, especially *MYBPC3-A31P* mutations in cats.

This is the first study applying a LAMP coupled with an LFD test for screening *MYBPC3-A31P* mutations. Our findings demonstrated that *A31P*-LAMP associated with the LFD test can distinguish between wild-type cats and mutant cats with the *MYBPC3-A31P* mutation (Figure 6). Nevertheless, this technique has some limitations due to its inability to differentiate between homozygous and heterozygous mutation carriers.

After successfully developing a specific design of *A31P*-LAMP, the *A31P*-LAMP amplification was optimized for temperature and time (Figure 5). We found that the designed *A31P*-LAMP can be amplified in wide ranges of temperatures and with various equipment, such as thermocyclers, heated blocks, and water baths. This is consistent with a previous study, where the researchers developed a LAMP for detecting the *Mycobacterium leprae* pathogen that causes Hansen's disease. The researchers were able to conduct LAMP amplification in both a heated block and a water bath (29). Moreover, in this study, the average time to perform the *MYBPC3-A31P* test with LAMP was ~110 min, including the process of DNA extraction, DNA amplification, and SNP detection. These data indicated that the designed *A31P*-LAMP required less time than other methods for SNP detection, such as PCR with DNA sequencing. PCR with DNA sequencing takes a long time due to the numerous steps, including DNA extraction, PCR, purification of PCR products, and DNA sequencing methods (30).

The designed *A31P*-LAMP can be clinically utilized as a rapid assay for *MYBPC3-A31P* mutation detection. To reduce the SNP detection time, in future studies, DNA from various samples, such as blood, dry blood spots (DBSs), hair follicles, or buccal swaps, will be extracted with sodium hydroxide (NaOH). This method can save time for DNA extraction owing to its simple DNA extraction procedure (31).

The LFD test is a widely accepted technique for detecting LAMP products. Following numerous lines of previous evidence, a LAMP assay combined with an LFD test was initially used for detecting several diseases not only in animals but also in humans. For instance, LAMP with the LFD test has been reported for use in the detection of the hepatitis B virus in human fields (32). In this publication, the LAMP products were detected by agarose gel electrophoresis, LFD tests, and gold nanoparticles (AuNPs). The three methods had the same accuracy for hepatitis B virus detection. In humans infected with *Plasmodium falciparum*, LAMP with an LFD for specific SNP detection (*Pf* SNP-LAMP-LFD) was applied to investigate malarial disease. It has been reported that malarial disease is associated with antifolate molecular resistance to manage antimalarial drug strategies (33).

LAMP combined with LFD has been applied to detect hepatopancreatic parvovirus in shrimp (34). They documented that this method is more sensitive than one-step PCR with agarose gel electrophoresis due to the lower DNA concentration. In terms of the objective of this study, the combination of LAMP with the LFD test had superior sensitivity for the detection of the designed LAMP product compared with agarose gel electrophoresis at 0.00001 ng/μl (Figure 7). Based on these promising results, the designed *A31P*-LAMP coupled with the LFD test is a reliable assay for *MYBPC3-A31P* mutation screening. Similar to the previous study (35), our results showed that the LAMP assay only took 110 min, this method provides on-site detection of a pathogen without requiring complex equipment. The LAMP assay is a rapid, simple, and sensitive test. Therefore, the *A31P*-LAMP assay could be more suitable as a screening point-of-care test for detecting the *MYBPC3-A31P* mutation in Maine Coon cats than the conventional PCR methods. In our pilot study, only 7/55 cats (12.73%) were diagnosed using the *A31P*-LAMP assay coupled with the LFD test, which was a limitation. For future work, it will be useful to increase the number of cats tested; however, we still gained valuable results by using the *A31P*-LAMP-LFD test in this study. Another limitation was that we did not investigate the efficacy of diagnostic examinations, such as the sensitivity and specificity, for *MYBPC3-A31P* diagnosis with the *A31P*-LAMP assay coupled with the LFD test. This should be further investigated in a larger population of Maine Coon cats.

Novel methods for genetic testing of the *MYBPC3-A31P* mutation play a crucial role in reducing the effects of familial HCM in Maine Coon cats (26, 36). According to the American College of Veterinary Internal Medicine (ACVIM) consensus statement guidelines for the classification, diagnosis, and management of cardiomyopathies in cats, *MYBPC3-A31P* screening is suggested to decrease the abnormal gene pool and reduce HCM development in Maine Coon cats (26, 36). In this study, the *A31P*-LAMP method coupled with the LFD test is a rapid diagnostic tool for detecting *MYBPC3-A31P* mutations.

## Conclusion

Loop-mediated isothermal amplification for the *MYBPC3-A31P* mutation (*A31P*-LAMP) coupled with an LFD test has enormous potential for screening the crucial SNP mutation of the sarcomeric protein in Maine Coon cats with HCM. Due to its high sensitivity and simple evaluation, this technique can be readily applied for on-site routine examinations in pedigree breeding in cats.

## Data Availability Statement

The datasets presented in this study can be found in online repositories. The names of the repository/repositories and accession number(s) can be found in the article/supplementary material.

## Ethics Statement

The animal study was reviewed and approved by Kasetsart University Institutional Animal Care and Use Committee, Kasetsart University, Bangkok, Thailand (Approval Number: ACKU 62-VET-059). Written informed consent was obtained from the owners for the participation of their animals in this study.

## Author Contributions

SP and PS designed the study and wrote the original manuscript and prepared figures. SP, KD, and PS collected all samples and analyzed and interpreted the data. PS performed the experiments of genotyping and LAMP with LFD. SP finalized the manuscript. All authors have read and approved the final version of the manuscript.

## Funding

This research project was supported by the National Research Council of Thailand (NRCT): NRCT5-RGJ63002-035.

## Conflict of Interest

The research was conducted with a patent application (ID: 2103003416). The authors declare that the research was conducted in the absence of any commercial or financial relationships that could be construed as a potential conflict of interest.

## Publisher's Note

All claims expressed in this article are solely those of the authors and do not necessarily represent those of their affiliated organizations, or those of the publisher, the editors and the reviewers. Any product that may be evaluated in this article, or claim that may be made by its manufacturer, is not guaranteed or endorsed by the publisher.
